# Optimization of X-ray Investigations in Dentistry Using Optical Coherence Tomography

**DOI:** 10.3390/s21134554

**Published:** 2021-07-02

**Authors:** Ralph-Alexandru Erdelyi, Virgil-Florin Duma, Cosmin Sinescu, George Mihai Dobre, Adrian Bradu, Adrian Podoleanu

**Affiliations:** 1 Doctoral School, Polytechnic University of Timisoara, 1 Mihai Viteazu Ave., 300222 Timisoara, Romania; ralph.erdelyi@student.upt.ro; 2 3OM Optomechatronics Group, Aurel Vlaicu University of Arad, 77 Revolutiei Ave., 310130 Arad, Romania; 3 Research Center in Dental Medicine Using Conventional and Alternative Technologies, School of Dental Medicine, “Victor Babes” University of Medicine and Pharmacy of Timisoara, 9 Revolutiei 1989 Ave., 300070 Timisoara, Romania; minosinescu@gmail.com; 4 Applied Optics Group, School of Physics, University of Kent, Canterbury CT2 7NR, UK; gd@kent.ac.uk (G.M.D.); a.bradu@kent.ac.uk (A.B.); a.g.h.podoleanu@kent.ac.uk (A.P.)

**Keywords:** dental imaging, radiography, Optical Coherence Tomography (OCT), three-dimensional (3D) Cone Beam Computed Tomography (CBCT), image characteristics, radiation dose

## Abstract

The most common imaging technique for dental diagnoses and treatment monitoring is X-ray imaging, which evolved from the first intraoral radiographs to high-quality three-dimensional (3D) Cone Beam Computed Tomography (CBCT). Other imaging techniques have shown potential, such as Optical Coherence Tomography (OCT). We have recently reported on the boundaries of these two types of techniques, regarding. the dental fields where each one is more appropriate or where they should be both used. The aim of the present study is to explore the unique capabilities of the OCT technique to optimize X-ray units imaging (i.e., in terms of image resolution, radiation dose, or contrast). Two types of commercially available and widely used X-ray units are considered. To adjust their parameters, a protocol is developed to employ OCT images of dental conditions that are documented on high (i.e., less than 10 μm) resolution OCT images (both B-scans/cross sections and 3D reconstructions) but are hardly identified on the 200 to 75 μm resolution panoramic or CBCT radiographs. The optimized calibration of the X-ray unit includes choosing appropriate values for the anode voltage and current intensity of the X-ray tube, as well as the patient’s positioning, in order to reach the highest possible X-rays resolution at a radiation dose that is safe for the patient. The optimization protocol is developed in vitro on OCT images of extracted teeth and is further applied in vivo for each type of dental investigation. Optimized radiographic results are compared with un-optimized previously performed radiographs. Also, we show that OCT can permit a rigorous comparison between two (types of) X-ray units. In conclusion, high-quality dental images are possible using low radiation doses if an optimized protocol, developed using OCT, is applied for each type of dental investigation. Also, there are situations when the X-ray technology has drawbacks for dental diagnosis or treatment assessment. In such situations, OCT proves capable to provide qualitative images.

## 1. Introduction

The discovery of X-rays in 1895 by Wilhelm Conrad Roentgen is considered to mark the beginning of medical imaging [1]. Since then, the techniques have improved continuously in the last two decades. One may consider in this respect, for example, the quality differences between the first radiographs and today’s 3D CBCTs [2]. Numerous other imaging techniques have been developed for dental medicine, but radiography has remained its most common investigation tool. Therefore, improving X-rays techniques is potentially of high impact due to their wide usage. The question is: how much room is there left for such improvements? Also, how could they be achieved?

To respond to such questions, we must first observe that all X-ray units have the same structure. They consist of an X-ray tube, a sensor, and a PC that processes data. Today most possible improvements in X-rays-based medical imaging techniques rely on increasing the sensitivity of the sensors.

Thus, the first evolutionary step for X-ray detectors for dental imaging has been from photographic films to photo-stimulable-phosphor-plates (PSPs) [3]. This came along development of additional equipment for converting data from PSP into digital. In a second evolutionary step digital sensors fully replaced films, providing several benefits such as time saving, post-processing tools, and better image quality.

Today, cutting-edge X-ray dental units have digital sensors capable of providing high-quality images [4,5,6,7,8]. The difference between units available on the market is made by the characteristics of the sensors, such as spatial resolution or contrast. The most utilized types of digital detectors in dental imaging are charge-coupled devices (CCDs), complementary metal oxide-semiconductors (CMOS), and flat panel sensors [9].

Because X-rays consist of high-energy electromagnetic radiation, they can ionize atoms and disrupt molecular bonds [10]. In this respect, regulations have been set, based on the As Low As Reasonably Achievable (ALARA) protocol. In consequence, all X-ray units must be properly utilized, with an optimized workflow to achieve the best possible image quality with the smallest radiation dose [11,12]. This means that one cannot increase resolution, for example, by improving functional parameters of the X-ray tube, because the radiation dose must be kept to a minimum. Therefore, a trade-off must be reached between contradictory requirements. The question is: how to optimally achieve such a trade-off?

To respond to this question, the aim of this work is to explore innovative ways of optimizing the operation of (already high-performance) commercially available X-ray units. Two types of such high-end units are used for this purpose. The ALARA protocol is considered along with the sensor performance. Characteristics of X-ray images such as resolution, contrast, sharpness, or artefacts are adjusted via a calibration protocol involving an alternative imaging technique, Optical Coherence Tomography (OCT) [13,14,15]. OCT is based on low coherence interferometry that uses near infrared laser radiation. While X-ray images show the spatial distributions of X-ray absorption, OCT images show the spatial distribution of differences in refractive indices. OCT is non-invasive and has the advantage of better than 10 μm axial resolution in tissue [15,16], at least 10 times better than resolution achievable using X-rays imaging. In the last decade handheld OCT scanning probes have been developed, for in vivo investigation of eye [16], skin [17], other body parts [18], as well as dedicated to the oral cavity [19,20,21]. Another promising direction of research refers to the study of dental materials, as OCT can provide optical cross-section capability for their non-destructive testing [22,23,24,25,26,27]. However, despite its advantages, OCT cannot fully replace radiography, due to its limited penetration depth of only up to 2 mm in tissue.

In a recent study we explored the boundaries between OCT and the common radiography when applied to maxillo-facial medical imaging [28]. The study concluded that dental radiography and OCT can complement each other in assessing the oral cavity, with certain areas where only one of the above techniques would be applicable in diagnosing and monitoring clinical aspects, but also with areas where the two techniques may validate each other. The aim of the present study is to use the much higher resolution technique, OCT, in improving the imaging performance of the most common technique, radiography.

Such an approach can impact the practice of the X-ray imaging, widely used by medical doctors to provide suitable treatments and monitor the evolution and outcomes of patients. We also hope that this study will contribute to more acceptance of OCT in common dental practice [29,30,31,32,33], particularly as OCT is a technology already used on a daily basis in ophthalmology [34], dermatology [35], and endoscopy [36].

## 2. Materials and Methods

**Figure 1:**
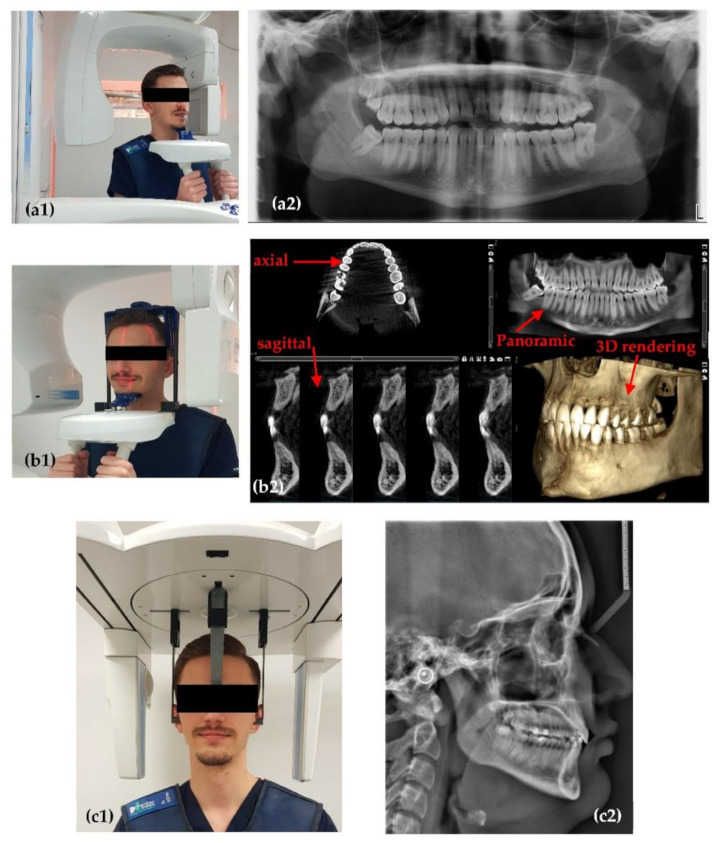
Example of the Planmeca setup for (**a1**) panoramic, (**b1**) 3D CBCT, and (**c1**) cephalometric investigations (of a healthy member of the staff). Obtained images: (**a2**) panoramic; (**b2**) 3D CBCT images, with axial, sagittal, and panoramic views, as well as a 3D reconstruction; (**c2**) cephalometric.

The X-ray imaging in this study is performed in the Dental Experts Clinic (Timisoara, Romania), using a ProMax 3D X-ray unit (Planmeca, Helsinki, Finland, Figure 1), as well as in other dental imaging clinics in Romania, that are using Soredex Cranex 3D X-ray units (KaVo Kerr, Brea, CA, USA). The OCT investigations are performed in the Laboratory of Optomechatronics and Biomedical Photonics of the “Aurel Vlaicu” University of Arad. The study is approved by The Ethical Commission of the Clinic, following the Ethical protocol, with the Approval 178/31.08.2020, and it is carried out according to the Declaration of Helsinki. An informed consent is submitted to all enrolled patients.

### 2.1. Planmeca ProMax 3D

The first X-ray unit used in this study is a Planmeca ProMax 3D system (Figure 1), equipped with a Toshiba X-ray tube (Toshiba Electron Tubes & Devices Co., Ltd., Otawara, Japan) and a Flat Panel Detector (Planmeca) based on CMOS sensors. Alongside the X-ray unit there is also a workstation and a PC for image reconstruction. The PC is gathering, transforming, and transmitting data from the X-ray unit sensor to the workstation equipped with the Romexis software (Planmeca). This dedicated software has specialized tools to help clinicians to enhance the raw images processed by the reconstruction PC, as well as to measure or analyze different aspects observed on the obtained images. Alongside the standard image reconstruction algorithm, the X-ray system is equipped with additional specialized algorithms for removing artefacts produced by the patient’s movements.

There are several possible dental images that can be obtained with this X-ray unit: panoramic, cephalometric, sinus, or 3D CBCT. Three such examples are presented in Figure 1. For each of them there is a standard protocol to operate the X-ray unit. Users are allowed to change parameters of the X-ray tube and sensor sensitivity alongside the exposure time. The X-ray tube characteristics are: focal spot size, X-ray filtration, current intensity (mA), anode voltage (kV), and exposure time (s). The focal spot size in this case is 0.5 × 0.5 mm^2^, the total filtration is performed with a 2.5 mm Al layer for two-dimensional (2D) images, and with a 2.5 mm Al layer plus a 0.5 mm Cu layer for 3D images. The anode voltage of the X-ray tube is in the range of 60 to 90 kV, while the current intensity is in the range of 1 to 16 mA.

Sensor characteristics of this X-ray unit that are important for the final image are: sensor dimensions (110 mm width and 80 mm high), sensitivity (low dose, normal, high definition, high resolution, and endo), and segmental possibilities, i.e., CBCT (cylindrical) volumes have a base diameter (mm) × height (mm) equal to: 80 × 80; 50 × 50; 110 × 50.

### 2.2. Soredex Cranex 3D

**Figure 2:**
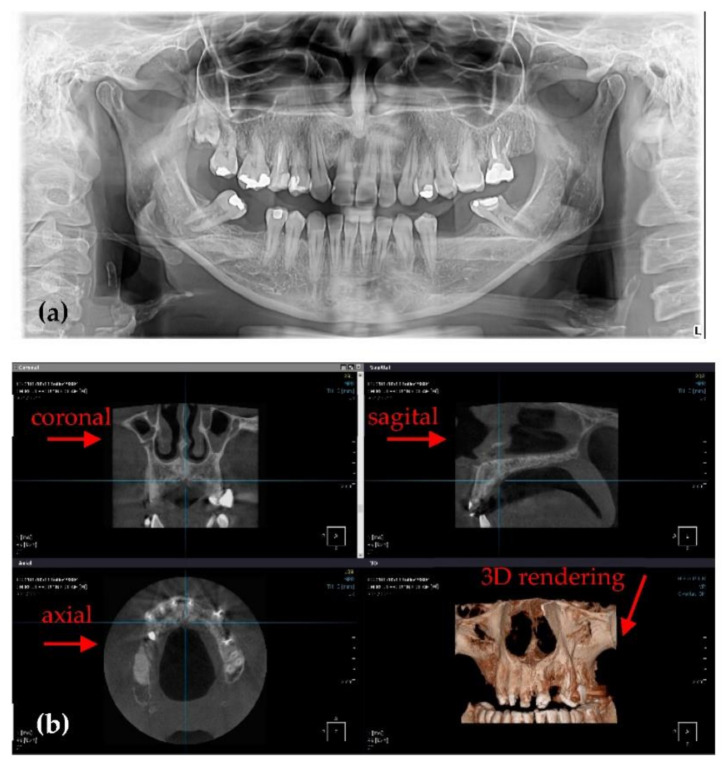
Examples of images obtained with Soredex setups (for a healthy member of the clinic’s staff): (**a**) panoramic and (**b**) 3D CBCT, the latter showing coronal, sagittal, axial views, as well as a 3D volumetric reconstruction.

The second type of X-ray unit used in this study is a Soredex Cranex 3D (Figure 2), equipped with an X-ray generator with a focal spot of 0.5 mm, a minimum total filtration of radiation beam with a 3.2 mm Al layer, anode voltage of 57 to 90 kV, and anode current of 4 to 16 mA. The X-ray detector, similar to the Planmeca X-ray unit, is a Flat Panel Detector based on CMOS sensors. Two examples of images obtained with this system in the clinic are presented in Figure 2.

There are differences between the Planmeca and Soredex system in terms of the volume that can be chosen for 3D CBCTs, and also in voxel and pixel dimensions. The cylindrical volumes for 3D CBCT available with the Soredex X-ray unit are with a base diameter (mm) × height (mm) equal to: 50 × 50, 61 × 78, 78 × 78, 78 × 150, and 130 × 150. The scanning time is 10 to 40 s. The exposure time is only 1 to 9 s, because the radiation beam is pulsed towards the patient, while the X-ray tube is not working during the entire scanning process.

### 2.3. OCT System

An in-house developed Swept Source (SS)-OCT system, Master Slave (MS) enhanced is used [37]. The scope is to investigate several dental samples in order to provide higher-than-radiography resolution images to allow for a proper calibration of the X-ray imaging systems.

**Figure 3:**
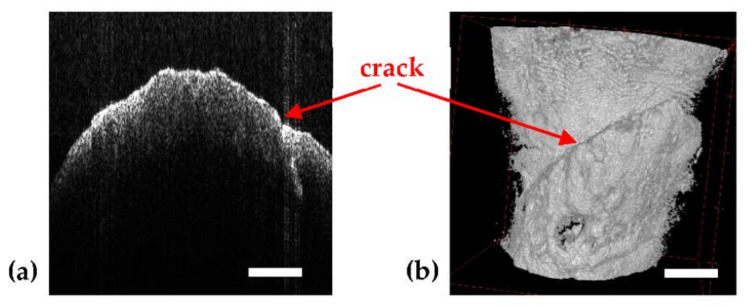
Example of a single OCT B-scan/optical cross section (**a**) and the corresponding 3D OCT image reconstruction (**b**), showing a crack in a tooth—example of OCT imaging showing the higher resolution capability compared to radiography, as approached in detail in the exploratory study in [28]. Scale: 1 mm.

The OCT system is presented in detail in [28]. It is centered at a wavelength of 1310 nm and uses a 2D dual axis galvanometer scanner (GS) for the lateral scanning of samples [38,39]. The maximum area of investigation with this system is 5 × 5 mm on the probe surface. The axial resolution provided by this OCT system is 10 µm in air, and the penetration depth in hard tissue is around 1.5 mm. During a complete scan, 500 B-scans/transversal cross-sections are obtained. Each one can be further analyzed, and measurements can be performed on it. Also, these 500 B-scans can be rendered into a 3D/volumetric reconstruction, as shown in the example in Figure 3.

### 2.4. The Concept of X-ray Imaging Optimization Using OCT

Theoretically, one could increase the resolution of X-ray images by simply increasing the anode voltage and current intensity of the X-ray tube, but this would imply an increase in radiation dose. This would be against the ALARA protocol. On the other hand, one cannot simply choose low settings of the X-ray tube, as this would mean a low image resolution, therefore the scope of the technique (i.e., diagnosis or treatment monitoring) would not be reached.

Therefore, a trade-off (i.e., an optimization process) is necessary but the question is where (and how) to set the limits for X-ray tube settings that can provide enough resolution for the medical scope but is able to keep the radiation dose as low as possible to protect the patient. Several aspects have to be clarified to design such an optimization process of an X-ray unit:

(1) An adjustment of X-ray tube and unit parameters cannot be done on patients (i.e., experimenting on them); it has to be carried out in vitro. Optimal settings thus determined could be then applied on patients. This logical sequence is used in the protocol to be developed in this work.
(2) An essential question is: what method to employ for an X-ray system calibration? It must provide better and, ideally, higher-order (from a metrological point of view) resolution images than radiography but related to the same targets/samples. Then, the X-ray tube settings could be adjusted to match the radiographic results with those of the “calibration” method, following an appropriate metrological approach.
(3) Finally, what type of higher-resolution system would be able to serve for such a calibration process? It has to be an imaging system, as devices used clinically for visual observation cannot allow for this planned calibration. Regarding imaging systems, all types of Computed Tomography (CT), including micro-CT are expensive and therefore out of reach of common dental practices, even of dental clinics, that would not invest in such equipment. The same cost limitation refers to high-resolution systems such as Scanning Electron Microscopy (SEM). On the other hand, dedicated devices for the oral cavity, such as the Diagnocam (KaVo Kerr, Brea, CA, USA) or the VistaCam (Dürr Dental SE, Bietigheim-Bissingen, Germany) may provide resolutions similar to those of radiographs (but only for certain areas that they are capable to investigate), therefore they are not a higher-order resolution method (on the metrological chain).

In conclusion, the only technique that satisfies the criteria of higher resolution, reasonable cost of the equipment, but also ease-of-operation and in-depth imaging (unlike confocal microscopy, for example) is OCT. It also has the advantages of non-invasive investigation and of the possibility to perform in vivo imaging, when necessary using dedicated handheld scanning probes [16,17,18,19,20]. As a plus, it benefits from mobile units [18] and, as studied in detail in [28], it proves to be complementary to radiography for diagnosis, treatment monitoring and assessment. Therefore, there is a clear motivation for dental clinics to utilize such systems: using OCT for the calibration of X-ray units just adds to their range of dental applications.

One may also remark that X-ray imaging is not able to resolve the features that OCT can do even if fully optimized. While this is correct for a range of investigations, as we studied for example in [40], based on the quantitative assessments performed in [28] we demonstrated that radiography can spot relevant details such as small cavities, but cannot correctly measure them like OCT can (i.e., errors around 50% are possible even with CBCT). This shows that OCT can serve as a calibration tool for X-ray images that resolve such relevant dental conditions.

### 2.5. OCT versus Radiography

**Figure 4:**
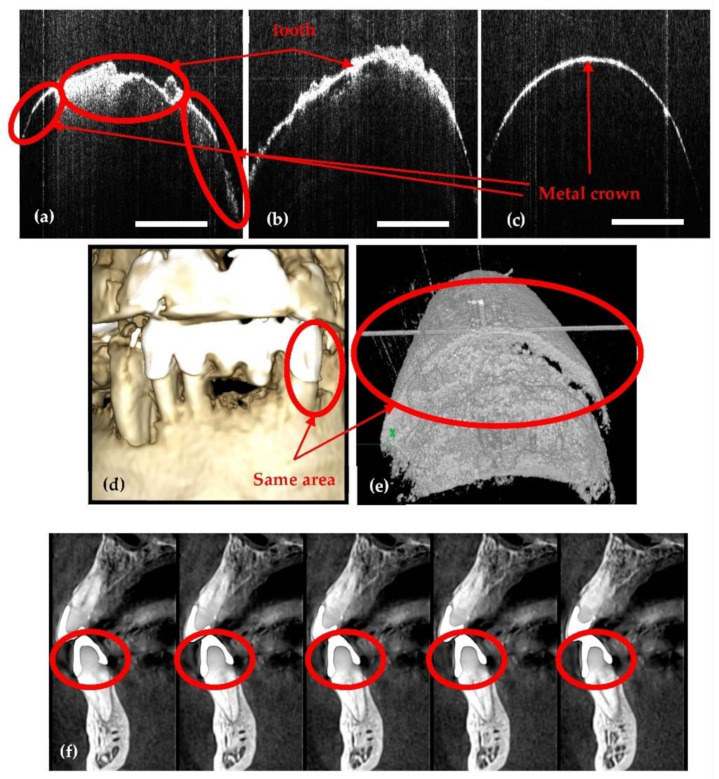
An example where OCT spots dental details near metal crowns. (**a**–**c**) OCT B-scans; (**d**) 3D rendering of CBCT; (**e**) OCT 3D reconstruction; (**f**) 3D CBCT sagittal view of the tooth. Scale: 1 mm.

Because OCT employs IR laser radiation, it cannot provide images beneath metal surfaces (e.g., of metal crowns as those shown in Figure 4), but it can provide clear images in their vicinity (Figure 4a–e). In contrast, 3D CBCT cannot achieve such images, because of artifacts produced due to metal, as shown in the example in Figure 4d,f. In time, if secondary cavities appear, for example, these cavities can be observed on CBCT images only when they become large enough and surpass the dimensions of artifacts.

**Figure 5:**
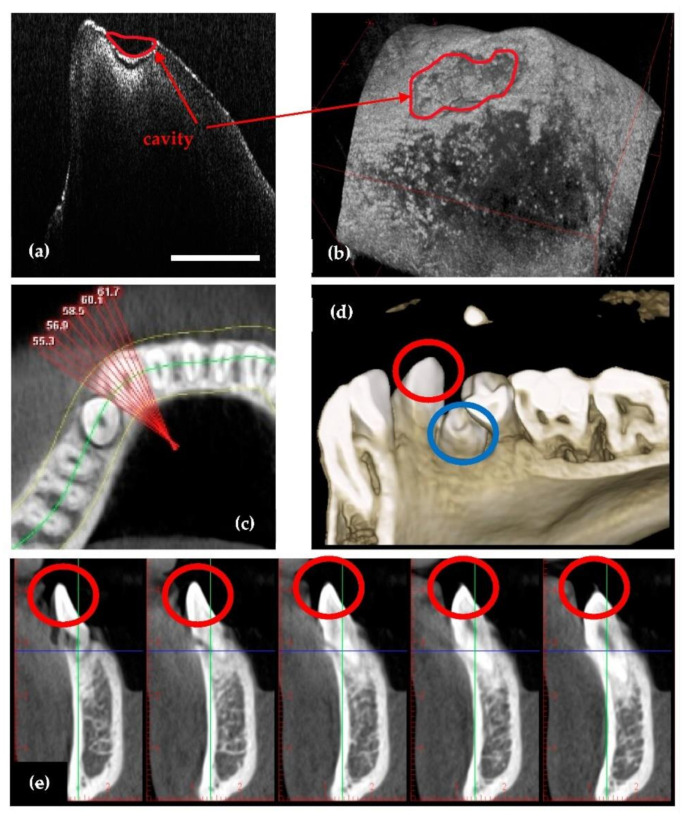
Example of a case where OCT spots a small cavity on the enamel level of the tooth: (**a**) OCT B-scan; (**b**) OCT 3D reconstruction; (**c**) 3D CBCT axial view of the tooth; (**d**) 3D rendering of CBCT; (**e**) 3D CBCT sagittal view of the tooth (the latter taken at different, successive depths into the hard tissue). Scale: 1 mm.

**Figure 6:**
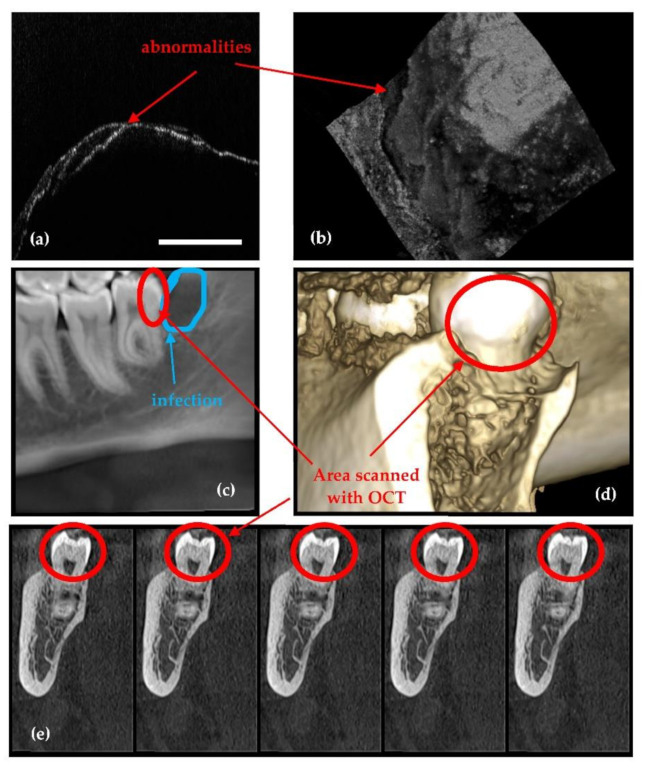
Example where OCT spots abnormalities on the enamel and dentine level of the tooth. (**a**) OCT B-scan; (**b**) OCT 3D reconstruction; (**c**) 3D CBCT axial view of the tooth; (**d**) 3D rendering of CBCT; (**e**) 3D CBCT sagittal view of the tooth (the latter taken at different, successive depths into the hard tissue). Scale: 1 mm.

Also, there are cases where dental radiographs cannot be used for diagnoses or treatment because of limitations such as: missing details in small cavities (Figure 5), abnormalities (i.e., cracks or deformations) of dentine or enamel (Figure 6), or dental issues near metal crowns (Figure 4). As discussed in our previous study [28], to cover all such cases of dental imaging, OCT proves to be the appropriate solution. A classification of dental medicine conditions with regard to the applicability of one imaging technique or the other (i.e., X-ray imaging or OCT) highlighted that each of them have certain domains of aplicability, while these domains overlap for certain medical conditions [28]. Roughly, OCT wins when resolution is required, while radiography wins where penetration depth is paramount.

In the case presented in Figure 5, the tooth marked with red must be extracted because an orthodontic treatment is mandatory, i.e., the extracted tooth has been blocking the tooth marked with blue in Figure 5d. On the radiographs it can be observed that the extracted tooth looks healthy, as no cavities are visible. After the extraction, the tooth is investigated with OCT, and a small cavity is observed on the enamel level. OCT resolution made possible even the assessment (i.e., exact measurement) of the cavity. Although this example refers to an extracted tooth, the capability of OCT to correctly assess and diagnose small cavities for in vivo investigations has been approached, as well [19,20,21,22].

The patient with the case presented in Figure 6 has an infection near the third molar, as shown in Figure 6c. Due to the massive infection visible behind it, the tooth has to be extracted. In addition, the doctor suspected that the dentine and enamel of this tooth was already damaged by the infection and the poor accessibility for cleaning. These abnormalities consist of dentine and enamel deformation, as well as superposed layers of dentine and enamel with random empty spaces between them. Thus, although the tooth looks healthy on the radiographs (Figure 6c–e), on the OCT images obtained after extracting the tooth for medical reasons abnormalities are visible both on the dentine and at the enamel level (Figure 6a,b). This can be best seen by comparing the images in Figure 6b,d, which are 3D reconstructions for OCT and CBCT, respectively. On 3D CBCT, the image resolution does not allow spotting small details of dental issues. This shows both the complementarity of the two techniques [28], as well as OCT capability to serve as a possible calibration technique for radiography, due to its higher axial resolution (10 µm compared to 150 µm for 3D CBCT resolution in these images).

## 3. Results and Discussion

The several necessary steps to develop the optimization imaging protocol are presented in this section. The scope is to obtain the highest possible quality of X-rays images with the smallest amount of radiation for the two considered commercially-available (and worldwide used) X-ray units. The possibility of using the higher resolution OCT as their calibration and validation technique (but also suitable for daily basis dentistry) is explored.

### 3.1. Optimized Protocol with OCT for X-ray Imaging Calibration. Panoramic Radiography

The OCT system has an axial resolution of 10 µm and both panoramic and 3D CBCT X-rays images have a resolution of 150 to 200 µm (both axial and radial) for daily basis usage. The highest resolution reached with a Planmeca X-ray unit is 75 µm for segmental 3D CBCT. Because OCT resolution is clearly superior to the one of any type of X-ray image, we compare OCT images to different X-ray images during the proposed optimization process. In other words, teeth are analyzed with both techniques and a dental issue that is spotted on both images is furthermore assessed with OCT. Thus, this identified dental clinical condition (for example a small cavity) is properly diagnosed using OCT. The same condition is then followed on X-rays images. The parameters of the X-rays unit (i.e., current intensity and anode voltage of the X-ray tube) are adjusted until the images of the dental details correspond as well as possible to the images retrieved with OCT. An assessment of the parameters of these images is then carried out, to confirm and quantify their improvement.

**Figure 7:**
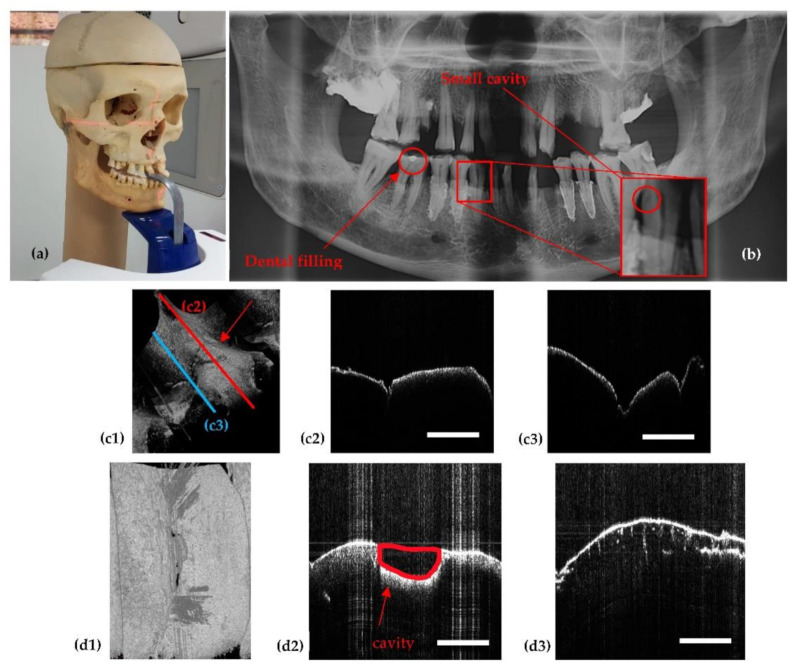
(**a**) The didactic human head used in the calibration process. (**b**) Panoramic radiograph illustrating the teeth that were further investigated with OCT. (**c1**) OCT volumetric reconstruction of the tooth with a small dental filling, marked in (b); (**c2**) B-scan/cross section corresponding to the position of the red line placed on the 3D view of the tooth in (c1); (**c3**) B-scan related to the blue line position in (c1). (**d1**) OCT volumetric reconstruction of a tooth that has a small cavity barely visible on the panoramic radiograph in (b); (**d2**) B-scan showing the cavity in (b); (**d3**) B-scan showing an additional condition of the tooth, i.e., demineralization (observed from the small cracks in the dentine). Scale: 1 mm.

The ionizing nature of X-ray radiation means that it can be harmful for living tissue. Therefore, as discussed in Section 2.4, we cannot test the different settings of the unit directly on patients. Thus, a didactic human head (Figure 7a) and several extracted teeth placed on this head are used in this study to develop the protocol, instead of living patients. This follows the procedure we first employed in [7], to comply with the ALARA protocol. However, in a clinical setting, a certain number of teeth, as used in [28] can be employed to develop the proposed protocol for another type of X-ray unit.

Figure 7c,d show examples of two considered dental details, a dental filling and a cavity (while the demineralization visible in Figure 7d3, although perfectly visible with OCT cannot be assessed with X-rays). The detailed OCT images assist with the optimization process of the X-ray unit. Thus, the dental details are followed on the X-ray image, such as the panoramic one in Figure 7b. One can observe that on both the OCT volumetric reconstruction and on its B-scans (Figure 7c,d) the dental filling and the small cavity, respectively, can be easily seen, while on the X-ray image in Figure 7b, they are barely visible.

For each type of radiograph (i.e., panoramic, cephalometric, and 3D CBCT), the proposed protocol starts with the smallest settings available for the values of anode voltage and current intensity of the X-ray tube. The energy and penetrability of the generated X-ray beam are proportional with the anode voltage, and the amount of radiation in the beam is proportional with the current intensity that is passing through the anode filament. A higher value of the anode voltage is providing a lower contrast of the image, while a higher value of the current intensity means a higher level of radiation, which is better to avoid.

**Figure 8:**
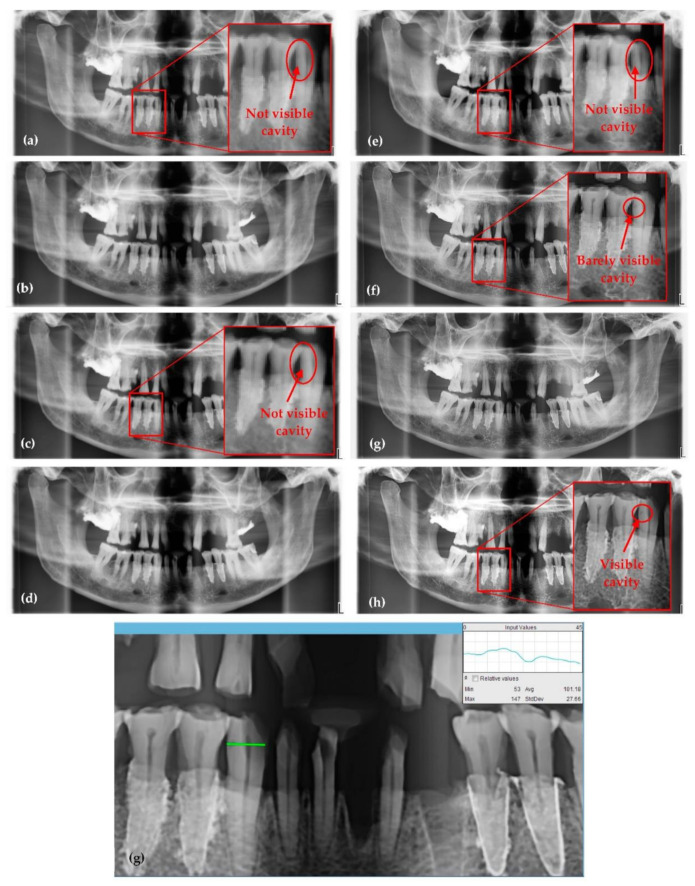
(**a**–**h**) Panoramic radiographs obtained during the optimization process. (**g**) Area with detail (small cavity) followed on each radiography image using OCT images as reference. The settings of the X-ray unit to perform each image and the quality parameters around the small cavity are provided in Table 1. The quality parameters of the entire radiographic images are compared in Table 2.

The results in Figure 8 are presented as a comparison between images obtained with the same X-ray unit before and while passing through the optimization process. The settings of the panoramic imaging before and during every step of the optimization process are provided, for each image in Figure 8, in Table 1. Thus, after a step-by-step increase of the values of the anode voltage and current intensity, it can be observed that the image quality increased, but the same happened to the radiation dose.

Therefore, ***to comply with the ALARA protocol, one has to choose the settings for which the image just passes a compromised threshold between a good and a high-quality image for both diagnosis and treatment.*** *Being guided by the precise OCT image, one can work on the parameters of the X-ray unit until fine details become visible as much as possible. This is the principle of the proposed (and performed) imaging* *optimization.* This procedure is presented further on in a following section using both X-ray units.

One can observe the progress made regarding the quality of the panoramic radiographs from Figure 8a–h, for the different values of voltage and current intensity presented in Table 1. The contrast, resolution, artifacts, as well as the level of overexposed and underexposed areas are improved from one panoramic radiograph to another, as it can be seen, but also quantified (Table 2).

Thus, the radiograph in Figure 8a is obtained with the smallest amount of radiation and the smallest possible values of the tube parameters, 60 kV and 1 mA, while the radiograph in Figure 8h is obtained with the values for 72 kV and 11 mA that (just) passed the threshold between a good and a high-quality panoramic radiograph. Planmeca X-ray units allow for the change in settings for anode voltage and current intensity (from the default setting of the machine). Even if only a few parameters are adjustable, they are sufficient to improve the quality of X-ray images:

(1) A higher value of anode voltage means higher energy of the X-ray beam, hence X-ray photons of shorter wavelengths. Therefore, the higher the voltage, the larger the differences in absorption of the radiation that passes through tissue (according to Lambert-Beer’s law), therefore more shades of gray appear in the images. At first sight this may seem a drawback but the increase in the number of shades of gray with sharp edges means more details on the image, which is advantageous for medical imaging. However, this voltage increase is limited, as explained, to keep the radiation dose at safe levels for the patient.
(2) The number of X-ray photons emitted in time depends on the current intensity that is heating the filament of the X-ray tube. This quantity is also known as the intensity of the X-ray beam or radiation exposure.

The step-by-step increase of voltage and of current intensity shown in Table 1 is coupled with an almost constant exposure time, but with a significant increase in radiation dose. Analyzing Table 2, it can be observed that the best levels of contrast (C) and contrast-to-noise-ratio (CNR) in the final radiograph in Figure 8h are obtained by a cumulated effect of the above adjustments. Even though there are small differences between the values of C (0.998 highest and 0.961 lowest) and CNR (3.069 lowest and 3.263 highest), clear differences between the highest quality image (Figure 8h) and the lowest one (Figure 8a) can be noticed.

To further enter in the details of the optimization process, the small dental cavity considered in Figure 7b is presented in several of the imaging steps shown in Figure 8, including in the area selected in Figure 8g. While from Figure 8a–e this cavity is not visible at all, it becomes barely visible in Figure 8f, and in Figure 8h it is clearly visible. To quantify strictly this aspect, in Table 1 the CNR is slightly decreasing with every step of the optimization, because only the tooth was selected, and there is not too much difference between the shades of gray regarding tooth, cavity, and nerve. However, the values of C are increasing significantly as the cavity appears clearer and clearer in the succesion of images in Figure 8.

From Table 1 and Table 2 one can see that in this optimization we do not have the contrast *C* as a function of intensity and voltage with a minimum or a maximum, such an extremum being the optimum. Instead, C (but the same discussion can also be carried out for *CNR*) increases continuously with the two X-ray tube parameters. Therefore, one does not come from a distal point, go through optimum and continue beyond towards lower levels of the parameter to be optimized. As this is actually an optimization with constraints, we do not reach a maximum and then go down in the level of that parameter (i.e., resolution, C, or CNR) but we reach an as good as possible level of the image (and, as demonstrated in Table 2, of image parameters) using OCT images as reference, and we do not go beyond that because this would mean further increasing the radiation dose.

A remark that must be done is that when one uses one or several dental conditions, observe them with OCT and adjust the X-tube parameters, then image parameters (e.g., C and CNR) around that feature are improved, but so does the entire X-ray image. One cannot say that the 2D image (or volume, for 3D CBCT) in other parts except around the considered features suffers because of parameters adjustment, as the X-ray machine provides a homogeneous imaging process.

As a necessary remark, all determined settings refer to the didactic human head, which does not have soft tissue to influence the results. Therefore, to apply these settings on humans, technicians performing radiographs must consider the patient’s anatomical characteristics. In practice this means that small adjustments must be applied in certain cases, specifically an increase or decrease of 1 to 2 kV, as well as of 1 to 2 mA for the anode voltage and current intensity, respectively, with regard to the values in this work. Such modifications are necessary in order to achieve optimized radiographies for patients, as exemplified in Section 3.4.

### 3.2. Optimized Protocol with OCT. 3D CBCT Calibration

For every type of radiography (i.e., panoramic, cephalometric, and 3D CBCT), suitable (and different) settings must be determined for the optimization. Therefore, after the above discussion on panoramic images, the most utilized case of CBCT investigations is considered in the following.

**Figure 9:**
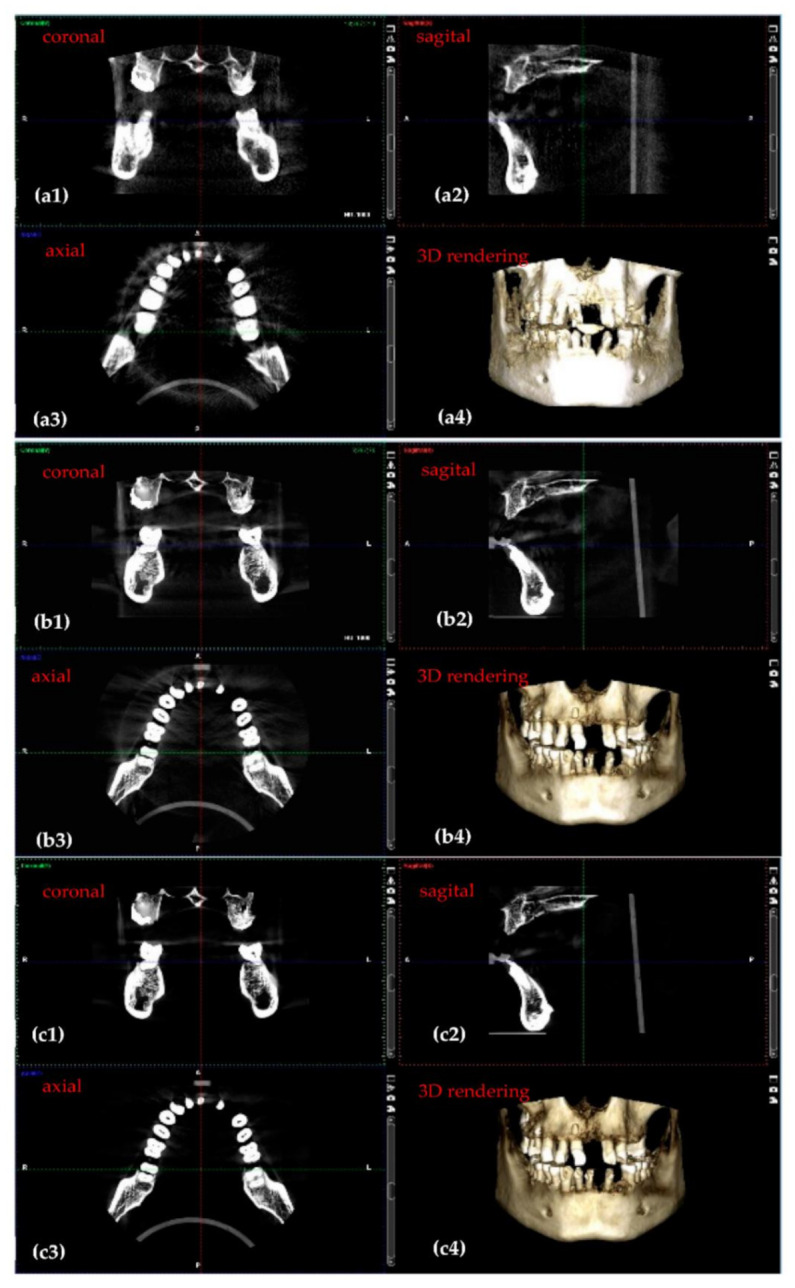
3D CBCT images obtained through the optimization process. The steps of this process include: (**a**) first 3D CBCT images with the smallest voltage and current values (**b**) an intermediary 3D CBCT; (**c**) the most high-quality 3D CBCT obtained. The four corresponding images in each panel represent: (**1**) coronal view; (**2**) sagittal view; (**3**) axial view; (**4**) 3D rendering.

Figure 9 presents 3D CBCT images obtained during the optimization process. The corresponding 3D CBCT settings before and during every step of this optimization process are presented in Table 3.

The improvement in resolution and quality of images can be seen by comparing Figure 9a–c. In this case, Figure 9b represents the threshold between a good and a high-quality 3D CBCT. Considering the fact that the sample does not have soft tissue (the human skull in Figure 7a is considered for this optimization, as well), the amount of radiation (i.e., the settings) used for the 3D CBCT in Figure 9b can be used with success for a child. For adults, the settings corresponding to Figure 9c were used further on with success on a daily basis activity in the dental clinic.

3D CBCT imaging followed the same protocol as in the case of panoramic radiographs. Both the images in Figure 9 and the values of the output parameters provided in Table 4 prove that the optimization is completed. Thus, the improvement in contrast, resolution, sharpness, and the level of detail from Figure 9a–c is confirmed in Table 4 regarding contrast: C is better for the final 3D CBCT (Figure 9c) than for the first one considered (Figure 9a). Thus, there is a difference of 0.15 between them (from the 0.82 highest contrast to the 0.67 lowest contrast). In common language, this improvement of contrast is pointed out on radiographies as ‘clean margins’.

As a remark, one cannot make a discussion (similar with the one in Figure 8 and Table 1) on values of C and CNR in the specific spots targeted on CBCT images during the optimization process carried out using OCT, because the Planmeca software does not provide values of I_max_, I_min_, and σ_0_ for local spots in 3D images.

### 3.3. Application of the Optimization Protocol on Patients (In Vivo)

The optimization process is considered to be completed once suitable settings for the X-ray unit are determined. Afterwards, these new settings can be used to investigate patients. Regarding ethical aspects, we must highlight that patients do not have to be informed that the X-ray unit will have different settings than the common ones, as the radiation doses do not exceed safety limits after the optimization process. Therefore, the new settings of the X-ray unit (i.e., the optimized radiography protocol) can be further on implemented for daily basis procedures.

**Figure 10:**
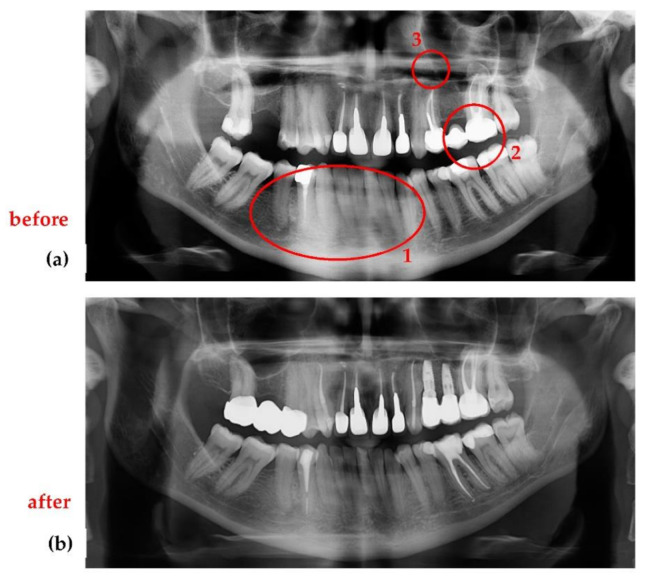
Panoramic radiographs performed on the same patient (L.C., female, 42 years old) (**a**) before and (**b**) after the optimization.

The following figures present several examples of similar types of examinations performed on the same patients, with optimized versus non-optimized protocols. In Figure 10, the flawed areas of a non-optimized panoramic radiograph are highlighted. They are not observed anymore on the panoramic radiograph performed after the X-ray unit optimization. Thus, on the panoramic radiograph in Figure 10a one can see overexposed areas on the mandible (i.e., white areas-zone 1). Also, the roots of these teeth cannot be accurately examined. The third molar from the third quadrant is overexposed (zone 2) and the first molar from the second quadrant appears like it has a mass (such as a cyst or fragmented bone) on its roots (zone 3). There is an issue caused by the small distance between the tooth and the sinus. If there were a cyst, it would be possible to spread the infection into the sinus, which should be avoided.

Therefore, the patient would be recommended to undergo other investigations (i.e., 3D CBCT or intraoral radiograph) to clarify issues raised by such an inconclusive panoramic radiography. After the optimization of the X-ray settings and after applying the necessary protocol to obtain high-quality radiographs with doses of X-ray radiation as low as possible, it can be seen on the panoramic radiograph in Figure 10b that all the bones and teeth have clean margins. Thus, the patient can be successfully analyzed, and she/he does not have to perform any other radiological investigations.

**Figure 11:**
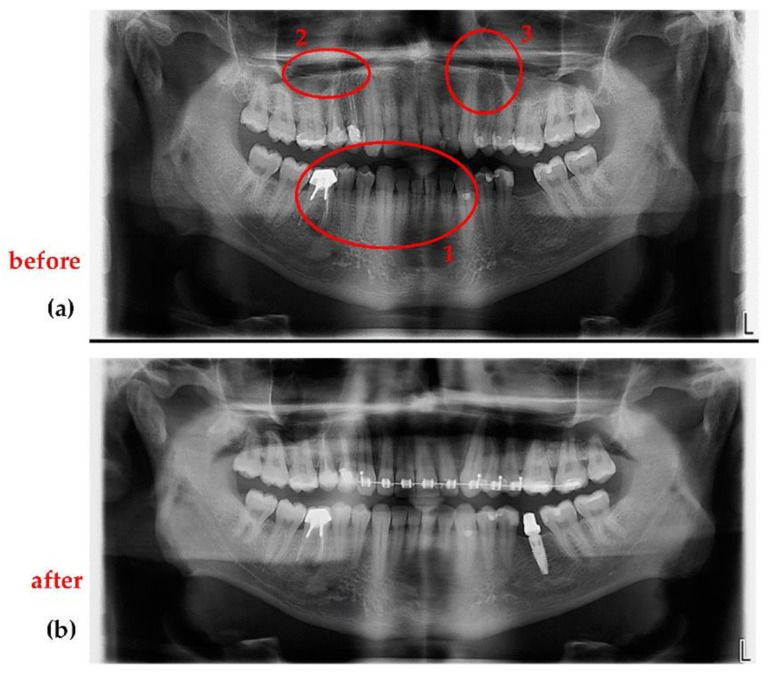
Panoramic radiographs performed on the same patient (F.C., male, 29 years old) (**a**) before and (**b**) after the optimization.

Figure 11 shows another example of two panoramic radiographs before and after the optimization of the X-ray unit. On the radiographs made before this optimization (Figure 11a) there are underexposed areas, as well as areas for which the sharpness and contrast is so low that features such as roots, root canals, or even clear margins of teeth are not visible (zone 1, for example). On the mandible, frontal teeth seem to be shorter than in reality(zone 2). On the maxillary, the threshold between teeth and sinus is barely visible on both quadrants. These issues are all corrected after optimization (Figure 11b).

In the case of panoramic radiographs, before and after parameter values are found to be close. This is the case of the voxel dimensions; anode voltage before the optimization was 68 kV and after the optimization is 72 kV; current intensity and exposure time are the same before and after the optimization. However, even though there are only small adjustments for the X-ray unit settings, the results are visible, with better quality of panoramic radiographs after in contrast to before optimization. However, for 3D CBCT the set differences are large, as discussed in the following.

**Figure 12:**
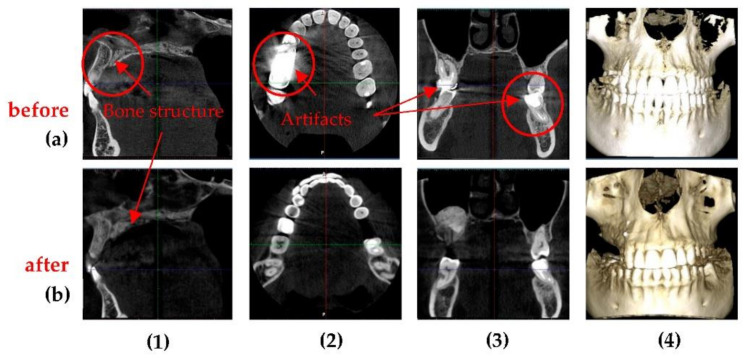
3D CBCT performed on the same patient (F.C., male, 29 years old): images (**a**) before and (**b**) after the optimization. Notations: (**1**) axial, (**2**) sagittal, (**3**) coronal, and (**4**) 3D rendering.

Figure 12 shows an example of a 3D CBCT made before and after optimization. It highlights other differences between the images obtained before and after optimization: (1) the bone structure is closer to reality on the optimized 3D CBCT; (2,3) the materials used for cavity filling having a high radio-opacity are not producing artifacts on the optimized images; (4) the resolution is better after optimization, as the dimension of the voxel side decreased from 200 to 150 µm.

**Figure 13:**
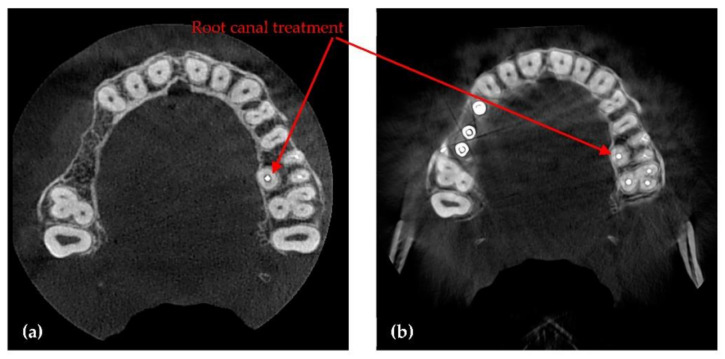
Example of an axial section (**a**) before and (**b**) after the optimization of the 3D CBCT imaging.

In the example in Figure 13, the root canal treatment of the same patient provides more details due to an increased resolution. In Figure 13a, the shape of the root canal treatment seems to be a square, while in Figure 13b one can observe its real shape. In addition, there are other clear differences between images obtained when investigating the same patient. Thus, before the optimization, the image does not have clear margins, while after the optimization, it does. Also, the bone density is misleading due to the size of the pixels. As in the previous example, in Figure 13a the pixel side is 200 µm, while in Figure 13b it is reduced to 150 µm (Table 5).

Several aspects should be highlighted in relation to the conclusions in Table 5. First, the voxel side is 25% smaller after the optimization process, which leads to a higher resolution. Second, even if the investigated volume is larger (i.e., a cylinder with the base diameter of 110 mm instead of 80 mm, with a height of 80 mm in both cases), the radiation dose is smaller: Dose Area Product (DAP) value after optimization is 6.9 mGy×cm^2^ instead of 11.7 mGy×cm^2^ before optimization. This is possible because the exposure time dropped from 12.057 s to 5.072 s when the optimized protocols were chosen.

### 3.4. Differences Between the Planmeca and the Soredex System

The optimization process must consider the specific X-ray unit. To this goal, Table 6 lists the most important parameters of both X-rays units used, determined for a daily basis investigation. Where a range of values is listed in Table 6, they refer to allowed variations depending on patient’s anatomical characteristics.

**Figure 14:**
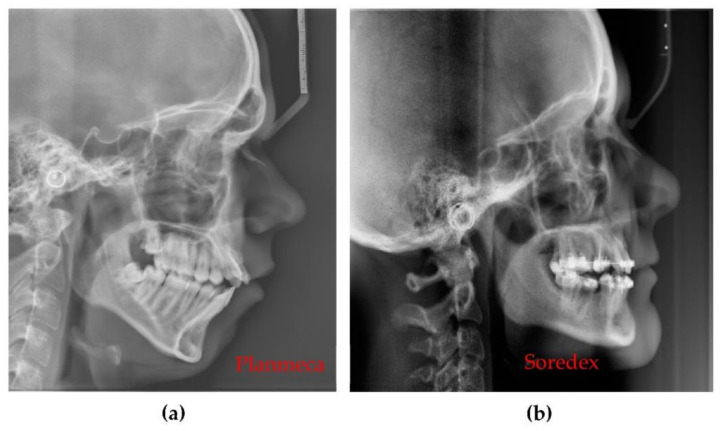
Cephalometric radiographs performed with (**a**) the Planmeca unit and with (**b**) the Soredex unit.

In addition, Planmeca and Soredex X-ray units have other settings that provide better resolution images, but with the drawback of a higher X-ray dose. Also, there are other types of radiographs that can be performed with each X-ray unit. Although for this study we focused on panoramic radiographs and (total, segmental and maxillary or mandible) 3D CBCT, in Figure 14a Cephalometric radiography is also shown.

**Figure 15:**
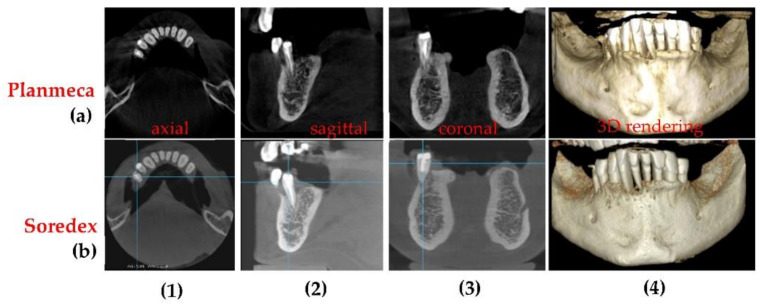
3D CBCT performed on the same patient (B.R., female, 53 years old) with (**a**) the Planmeca unit and with (**b**) the Soredex unit. Notations: (**1**) axial, (**2**) sagittal, (**3**) coronal, and (**4**) 3D rendering.

**Figure 16:**
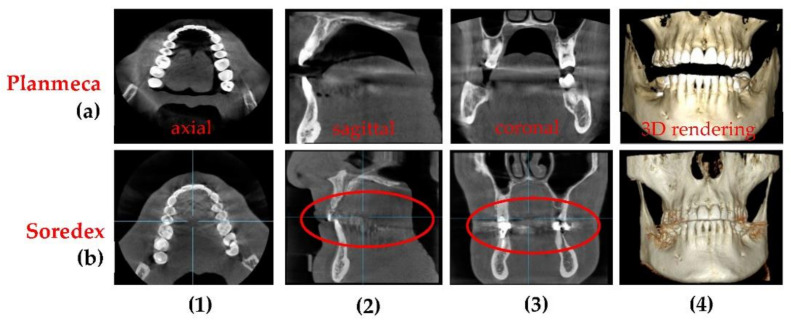
3D CBCT performed on the same patient (C.C, male, 48 years old) with (**a**) the Planmeca unit and with (**b**) the Soredex unit. Notations: (**1**) axial, (**2**) sagittal, (**3**) coronal, and (**4**) 3D rendering.

In general, Figure 14, Figure 15, Figure 16 present the same type of radiographs obtained with both types of X-ray units. They represent the cases of patients who came into the dental clinic with a 3D CBCT obtained with a Soredex unit. Unfortunately, these images were older than six months, therefore the patients had to be investigated again, and this time a Planmeca unit was used. This was the motive for new investigations for all the following patients exposed again to X-ray radiation.

An essential remark regarding all images is that the radiographs obtained with the Planmeca unit are performed after the optimization described in this work, while the radiographs obtained with the Soredex unit were performed for each considered patient with the specific protocol of other dental imaging clinics, prior to coming to the clinic where this work was carried out. The working protocols of the other clinics (i.e., with the Soredex unit) used the default settings of this X-ray unit, with non-optimized workflow and protocol.

Figure 14 shows that the optimized Planmeca Cephalometric radiograph is superior in terms of resolution, contrast, and quality to the non-optimized Soredex Cephalometric radiograph: there are small details such as the root canal that can only be observed on the image obtained with the Planmeca unit.

Figure 15 presents an example of two 3D CBCT performed on the same patient with the Planmeca and Soredex units. As expected, the advantage of a smaller voxel side (150 µm) of the optimized Planmeca unit allows to obtain a better radiograph than the non-optimized Soredex unit (characterized by a 200 µm voxel side). Also, Planmeca 3D CBCT images have the advantages of superior contrast compared to Soredex 3D CBCT images, as it can be easily observed on sagittal and coronal sections.

Figure 16 presents another case, with visible differences between two 3D CBCT in all images. From the axial sections one can observe that the optimized Planmeca image has more shades of gray, which means a better contrast and sharpness of the image. On the sagittal and coronal sections one can see that non-optimized 3D CBCT images made with Soredex have artifacts induced by the materials used for crowns and cavity filling (please see circled areas). 3D CBCT images made with Planmeca have almost no artifacts, while the existing ones (i.e., small sparkles on the exterior of the teeth crowns on the sagittal and coronal sections) do not influence the diagnosis or treatment. Another important aspect is that there are two protocols for positioning the patient, to obtain these 3D CBCT: in the case of Planmeca 3D CBCT, a minimum distance is needed between the patient’s maxillary and mandible, while for Soredex the patient needs to stand with mouth closed and with teeth in occlusion.

### 3.5. Remarks

**Figure 17:**
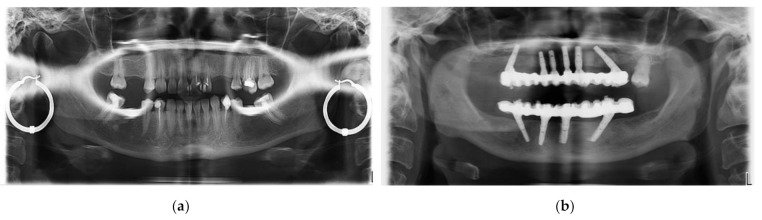
Imaging artefacts on panoramic radiographs: (**a**) in the case of a woman who cannot remove her earrings and (**b**) for a patient with ten implants and teeth reconstructions.

A few other aspects are worth discussing to facilitate the adoption of the optimization procedure into a daily dental imaging workflow:

(1) Different X-ray settings are needed for children, male or female patients (a different radiation dose is recommended to each of these three categories). Therefore, when the sample is changed, to achieve the optimum in X-ray imaging one must employ OCT again. However, a library of parameters can be obtained for different types of patients and for a specific machine.
(2) Following on from the previous point, human anatomical characteristics that can influence the radiography must be considered. For example, an overweight patient with a larger amount of fat tissue on mandible and maxillary must be exposed to a higher X-ray dose than a patient with normal weight.
(3) Radio opacity of dental materials used in previous treatments influence the quality of the radiographs. A patient with numerous metal crowns, for example, must be exposed to a lower radiation dose because otherwise artifacts may appear due to the high quantity of X-ray radiation absorbed by metals. This means that the values determined in this study might be different for other X-ray units, although the principle of the procedure remains the same. Thus, to achieve the best possible image, every X-ray unit should be calibrated and the best settings for anode voltage, current intensity, and exposure time should be obtained.
(4) Jewelry or any metal around the head or neck must be taken off, otherwise artifacts may appear on radiographs (Figure 17a). This is a general requirement, irrespective of the calibration procedure using OCT. On the other hand, implants and some materials used for dental crowns or dental fillings do not produce artifacts or sparkles around them on radiographs, as shown in the example in Figure 17b. This latter aspect must be considered during calibrations.
(5) The performances of X-ray units evolve continuously, including improvement in their radiation dose, to better comply with the ALARA protocol. Thus, radiation doses for 3D CBCT images made with Planmeca and Soredex units considered in this study are smaller than radiation doses found in studies carried out two decades ago, for example. Thus, in a study published in 2002 [44], the effective dose for a multi-slice CT was 740 µSv, the effective dose for Planmeca’s 3D CBCT was 86.4 µSv and for Soredex, 93.7 µSv. In another study, published in 2003 [45], the radiation doses were even higher: for a total 3D CBCT the effective dose was 2100 µSv, for maxilary 1400 µSv, for mandible 1320 µSv, for panoramic 10 µSv, and for intraoral radiographs 5 µSv. This remark is essential, as it points out that in the future, as the level of radiation doses may decrease, higher increases in other parameters, such as current intensity and voltage can be made. Therefore, such an OCT-based optimization protocol of X-ray imaging may become even more practical.
(6) Because it is using IR laser radiation, OCT does not penetrate metals, although studies of their roughness can be made [46] and, as shown in Figure 4, OCT can provide images near dental crowns, while 3D CBCT for example cannot achieve such images. Also, we have demonstrated that OCT can replace the gold standard of SEM in the study of metallic fractures [47,48]. Therefore, a subject of future work in our groups refers to OCT studies of metallic parts included in the oral cavity, for example dental implants.

## 4. Conclusions

We developed an optimization procedure applicable to the common X-ray radiography for dental medicine using OCT, which presents a much higher resolution. Two of the high-end commercially-available (and worldwide-used) X-ray imaging units were utilized, Planmeca ProMax 3D X-ray and Soredex Cranex 3D X-ray, both with state-of-the-art CMOS sensors. The principle of the optimization method is to obtain 10 μm axial resolution OCT images of dental details (such as cavities or dental fillings) and then to adjust the X-ray unit functional parameters (including anode voltage, current intensity, and patient position) until the observed detail becomes clear on the different types of radiographs (especially panoramic and CBCT). The increase in the X-ray tube parameters is made up to a threshold for which the X-ray image quality increases but the radiation level is kept to a minimum, to comply with the ALARA protocol.

The optimization procedure was developed on a didactic human head with extracted teeth and then was demonstrated on real-life patients, with comparisons between optimized and (previously made) not optimized radiographs. The output parameters of the imaging process, including contrast and contrast-to-noise ratio were assessed for every step of the optimization. Also, the developed procedure allowed for comparing performances of different X-rays imaging units. Finally, a comparison between images obtained with the two X-rays units and an in-house developed SS-OCT, MS enhanced OCT system is presented, as summarized in Table 7.

Furthermore, one can observe that there are no drawbacks on using OCT technique in addition to radiography. There are details that cannot be seen on radiographs, but they can be furthermore studied and assessed on OCT images. As demonstrated in our previous study [28], there is no competition between these two medical imaging techniques, even if there are medical conditions for which it is better to choose one method over another. However, in the end, it is convenient for a dental clinic to have both techniques available, for both purposes: (i) to be able to perform a correct and complete dental diagnose, treatments monitoring, and assessment in the complementarity of the two methods; (ii) to use OCT not necessary for imaging, but to aid choosing best parameters of X-ray units.

## Figures and Tables

**Table 1:** Panoramic settings before and during every step of the optimization process. Contrast (*C*) and contrast-to-noise-ratio (*CNR*) in the specific spots targeted on radiographs during the optimization process carried out using OCT.

Panoramic Radiographs (Figure 8)	Anode Voltage (kV)	Current Intensity (mA)	Exposure Time (s)	Radiation Dose (µSv)	C $=\frac{\left|I_{min}-I_{max}\right|}{I_{min}+I_{max}}$	CNR $=\frac{\left|I_{min}-I_{max}\right|}{\sigma_{0}}$
a	60	1	13.7	0.65	0.2	4.15
b	61	2	15	1.74	0.34	4.1
c	62	3.2	15	2.89	0.3	4
d	64	4	15	3.88	0.19	3.92
e	66	6.3	15	6.54	0.43	3.67
f	68	8	15	8.84	0.36	3.57
g	70	10	15	11.68	0.46	3.45
h	72	11	15	13.72	0.58	3.35

**Table 2:** Highest, lowest, and average values of pixel intensity *I*, *C*, *CNR*, and standard deviation of pixel intensity ($\sigma_{0}$ ), all related to the optimization process shown in Figure 8.

Panoramic Radiographs (Figure 8)	**$I_{min}\;$**	$I_{max}$	$I_{average}$ $=\frac{I_{min}+I_{max}}{2}$	$\sigma_{0}\;\left(\%\right)$	C $=\frac{\left|I_{min}-I_{max}\right|}{I_{min}+I_{max}}$	CNR $=\frac{\left|I_{min}-I_{max}\right|}{\sigma_{0}}$
a	3	3479	2104.47	1122.57	0.998	3.096
b	17	3377	2031.94	1094.54	0.989	3.069
c	20	3485	2012.62	1102.82	0.988	3.141
d	32	3317	1975.26	1060.52	0.980	3.097
e	31	3489	2106.08	1078.73	0.982	3.205
f	22	3456	2081.59	1056.53	0.987	3.250
g	70	3576	2172.58	1083.91	0.961	3.234
h	18	3593	2117.34	1095.44	0.990	3.263

**Table 3:** 3D CBCT setting before and during every step of the optimization process presented in Figure 9.

3D CBCT Radiographs (Figure 9)	Anode Voltage (kV)	Current Intensity (mA)	Exposure Time (s)	Radiation Dose (µSv)
a	60	1	4.95	1.25
b	75	8	5.09	25.87
c	90	14	5.08	86.37

**Table 4:** Highest, lowest, and average values of pixel intensity *I*, as well as *C*, all related to the optimization process presented in Figure 9.

3D CBCT (Figure 9)	$I_{min}$	$I_{max}$	$I_{average}=\frac{I_{min}+I_{max}}{2}$	$C=\frac{\left|I_{min}-I_{max}\right|}{I_{min}+I_{max}}$
a	552	2808	1680	0.67
b	302	2802	1552	0.8
c	296	3032	1664	0.82

**Table 5:** 3D CBCT setting before and after the optimization process.

	Diameter of Image Base (mm)	Image Height (mm)	Voxel Side (µm)	Anode Voltage (kV)	Current Intensity (mA)	Exposure Time (s)	$\mathrm{DAP}\;(mGy\times cm^{2})$
Before	Ø 80	80	200	84	14	12.057	1170
After	Ø 110	80	150	90	14	5.072	691

**Table 6:** Characteristic parameters of panoramic radiographs, as well as total, segmental, and maxillary or mandible 3D CBCT—for the two types of considered X-ray units.

Radiograph	Characteristics		Planmeca	Soredex
Panoramic	Anode voltage (kV)		68 to 73	70 to 75
	Current intensity (mA)		8 to 11	8 to 11
	Exposure time (s)		14.990	16
	DAP (mGy×cm^2^)		97 to 117	175 to 250
	Effective Dose (µSv)		7.8 to 9.2	14 to 20
	Pixel side (µm)		**127**	100
Total 3D CBCT	Anode voltage (kV)		90	85 to 90
	Current intensity (mA)		11 to 14	6 to 10
	Exposure time (s)		5	6 to 9
	DAP (mGy×cm^2^)		691*	749.5 **
	Effective Dose (µSv)		86.4*	93.7 **
	Voxel side (µm)		**150**	200
	Base diameter (mm)	of the investigated volume	110	150
	Height (mm)		80	80
Segmental 3D CBCT	Anode voltage (kV)		90	85 to 90
	Current intensity (mA)		11 to 14	6 to 10
	Exposure time (s)		5	6 to 9
	DAP (mGy×cm^2^)		329 *	140 to 300 **
	Effective Dose (µSv)		32.9 to 49.35	20 to 30 **
	Voxel side (µm)		**150**	200
	Base diameter (mm)	of the investigated volume	50	50
	Height (mm)		50	50
Maxillary/mandible 3D CBCT	Anode voltage (kV)		90	85 to 90
	Current intensity (mA)		11 to 14	6 to 10
	Exposure time (s)		5	6 to 9
	DAP (mGy×cm^2^)		429 *	400 ± 50 **
	Effective Dose (µSv)		42.9 to 64.35	40 ± to 60 ± 5 **
	Voxel side (µm)		**150**	200
	Base diameter (mm)	of the investigated volume	110	61
	Height (mm)		50	78

* Calculated for the provided level of kV and mA, with the remark that small deviations from these values can appear for different kV and mA levels. ** as obtained from different sources (for example from dental medical imaging centers equipped with similar type of units) and within the range of values documented in previous reports [41,42,43,44,45].

**Table 7:** Advantages and disadvantages of Planmeca and Soredex X-ray unit for panoramic radiographs, as well as total, segmental, and maxillary or mandible 3D CBCT. Comparison to OCT.

Method	Equipment	Advantages and Disadvantages
Panoramic	Planmeca and Soredex	Radiation dose is almost 50% smaller for Planmeca. Resolution is lower (127 µm) for Planmeca than for Soredex (100 µm). Images produced by Soredex have a lower contrast and sharpness even if they have better resolutions.
3D CBCT	Planmeca and Soredex	Smaller exposure time (5 versus 9 s), smaller radiation dose (with at least 10 µSv), and smaller voxel side (with 25%) for Planmeca, which means better resolution, contrast, and image quality. The covered volume is larger for Soredex.
OCT	SS-OCT	Better resolution, usually, around 10 µm axial (i.e., in depth), but it can be as low as 2 µm [17]. Lateral resolution (i.e., on the sample surface) is adjustable by galvanometer scanners programming; in this study it was set to 6 µm (corresponding to 500 B-scans for a scan length of 3 mm) or to 10 µm (for 500 B-scans per 5 mm). In contrast, the smallest achievable linear resolution (on each spatial direction) for 3D CBCT is 75 µm. Low penetration depth, but no ionizing radiation for OCT. The maximum volume scanned with OCT is 5 × 5 × 2 mm, while for radiography the volume corresponds at least to a cylinder with the base diameter of 50 mm and the height of 50 mm.

## Data Availability

Data are available on request from R.-A.E.
